# STEM, gender, and mental health: Understanding depression and anxiety in a national undergraduate sample

**DOI:** 10.1371/journal.pmen.0000364

**Published:** 2025-08-26

**Authors:** C. Jynx Pigart, Katherine A. Cohen, Riley McDanal, Jessica L. Schleider, Katelyn M. Cooper

**Affiliations:** 1 School of Life Sciences, Arizona State University, Tempe, Arizona, United States of America; 2 Department of Medical Social Sciences, Northwestern University, Evanston, Illinois, United States of America; 3 Department of Psychology, Stony Brook University, Stony Brook, New York, United States of America; University of Santo Tomas College of Science, PHILIPPINES

## Abstract

American universities have expressed increasing concern about the negative effects of mental health on undergraduates’ performance and persistence. Despite national calls to optimize mental health resources across universities, little effort has been made to identify which students struggle the most. Determining where university resources should be focused is crucial for maximizing their impact. STEM fields are hypothesized to exacerbate anxiety and depression among undergraduates because of their notoriously competitive and unwelcoming environments. Given that women are more likely to experience anxiety and depression in the general population, this study sought to assess to what extent the severity of anxiety and depression differ among cisgender men and women in STEM versus non-STEM fields. We analyzed data from 43,910 undergraduates representing 135 institutions who completed the Healthy Minds Study survey in the 2022–2023 academic year. Women self-reported more severe symptoms of anxiety and depression compared to men. STEM students self-reported more severe symptoms of depression, but not anxiety, compared to non-STEM students. The relationship between gender and major was not significant, meaning that the gender differences in anxiety and depression severity are neither magnified nor minimized in the context of STEM. However, women and STEM students disproportionately perceive that “anxiety/stress” affects their exam performance relative to men and non-STEM students, respectively. This study suggests that universities seeking to improve undergraduate mental health should recognize that college women and STEM students report more severe depression symptoms than their respective counterparts and that the impact of “anxiety/stress” on exam performance may disproportionately disadvantage women and STEM students.

## Introduction

In recent years, American universities have been plagued by a “mental health crisis,” owing to the remarkably high rates of anxiety and depression among undergraduates [1,2]. This crisis is hypothesized to be exacerbated for undergraduates in science, technology, engineering, and math (STEM) fields, compared to non-STEM fields, due to academically difficult and competitive STEM environments [3–5]. It is possible that women in undergraduate STEM fields in particular experience disproportionately poor mental health. Not only do women report higher rates of anxiety and depression compared to men, they also face unique challenges as a gender minority in most STEM fields [6–9]. These challenges can result in performance and retention gaps between women and men in college STEM majors [6–11]. However, no large-scale national studies have examined the mental health among women in STEM majors compared to women in non-STEM majors, men in STEM majors, and men in non-STEM majors. Further, it is unclear whether struggling with mental health has a differential impact on academic outcomes for students in these groups.

### Undergraduate mental health

Universities are increasingly concerned about the impact of anxiety and depression on undergraduates, as these conditions are linked to lower academic achievement and higher attrition rates [12]. Nearly 23% of American undergraduates report depression and 32% report anxiety [1]. The American Psychological Association defines anxiety as experiencing symptoms such as restlessness, fatigue, difficulty concentrating, irritability, and intense worry more days than not in a period of six months [13]. Depression encompasses experiencing symptoms such as feelings of hopelessness or sadness, diminished ability to think or concentrate, fatigue or loss of energy, and trouble sleeping every day for at least two weeks out of a year [14]. While national calls have sought to reduce undergraduate depression and anxiety, as well as optimize student mental health resources across universities [15–17], few efforts have been made to distinguish which university students struggle most with mental health and relatedly, where university efforts towards bettering student mental health should be focused to have the greatest impact.

### Mental health among undergraduates in STEM fields

STEM fields have been hypothesized to exacerbate symptoms of anxiety and depression [5,18–22] because of their notoriously chilly [23,24], competitive [3,4], and unwelcoming [7,25] environments. Additionally, recent research has suggested that undergraduate research experiences, which are more common in STEM fields than non-STEM fields [26], have the potential to worsen anxiety and depression, primarily when students engage in unstructured research experiences with little guidance and poor mentoring [18,27]. Further, individuals in STEM often experience “imposter syndrome”, occurring when an individual cannot internalize their successes and accomplishments, which is associated with feelings of self-doubt, anxiety, and depression [28–31]. Finally, STEM fields are known to provoke specific subject-related anxieties, such as math anxiety [32–34] and science anxiety [35–37]. Despite evidence suggesting that undergraduates pursuing STEM degrees may be particularly likely to report anxiety and depression compared to their non-STEM counterparts [5,7,18,20–24], there is little research documenting this trend. However, researchers who synthesized data collected across U.S. institutions from 2007-2013 found that natural science students and engineering students were no more likely to report anxiety or depression than their peers in the social sciences [17]. As such, a current analysis of student mental health among STEM and non-STEM undergraduates is needed.

### Mental health among women in STEM

Universities would benefit from delving deeper into how mental health varies among student demographic groups within these fields. Specifically, gender is a well-established predictor of mental health conditions; studies show that American women are more likely to report anxiety and depression compared to American men [38,39]. In addition, for undergraduate women, STEM fields are known to present unique challenges, which are either absent or less pronounced in non-STEM fields. For example, there are well-documented academic achievement gaps between undergraduate women and men in STEM fields; controlling for prior academic ability, men outperform women on STEM course exams [40]. Further, women are less likely to participate in STEM courses compared to men [40]. Not only do women report feeling less comfortable contributing to course discussions than men [41], they also contribute less frequently [40,42,43]. Undergraduate women majoring in STEM fields also report chilly or unwelcoming STEM environments [44,45] and lower self-efficacy, science identity, and belonging than undergraduate men in STEM [40]. It is difficult to disentangle the role that women’s mental health plays in their unique experiences within STEM fields because the relationship is likely reciprocal. For example, high levels of anxiety and depression may hinder women’s exam performance [8,46] and willingness to speak out in class [20,47–49]. However, poor performance on exams and discomfort when speaking out in class may further exacerbate women’s symptoms of anxiety and depression [47,48].

Considering that women are more likely to report anxiety and depression [38,39] combined with their unique experiences in STEM courses [40], it is unsurprising that women report worse mental health outcomes compared to men specifically in the context of college science courses [50–52]. However, what remains undocumented is whether the differing rates of anxiety and depression among men and women are magnified in STEM courses compared to non-STEM courses. In a sample of 318 undergraduate women from five higher education institutions, anxio-depressive symptoms were explored among male-dominated and gender-balanced balanced majors [53]. This study found that women in male-dominated STEM majors (e.g., engineering, physics, computer science) did not self-report significantly higher levels of anxio-depressive symptoms relative to women in gender-balanced non-STEM majors [53]. However, women in gender-balanced STEM majors (e.g., biological sciences, math) reported significantly worse anxio-depressive symptoms compared to women in gender-balanced non-STEM majors [53]. These findings underscore the complexity of the experience of women in STEM and non-STEM fields and emphasize the need for further research examining mental health by gender and major.

To date, no studies have investigated mental health disparities between undergraduate women and men across STEM fields collectively and at a national scale. Investigating whether mental health challenges are exacerbated in STEM fields stands to further our understanding of factors that discourage women from pursuing and persisting in STEM careers [54,55]. Additionally, information about gendered mental health disparities, or lack thereof, between STEM and non-STEM majors can guide colleges and universities in effectively delivering mental health resources to their undergraduates.

### Current study

To address this gap in the literature, we analyzed data from the Healthy Minds Study (HMS), a national, online survey distributed to college and university students across the U.S. We aimed to answer the following research questions:

**RQ1a:** To what extent does student-reported anxiety symptom severity differ among women in STEM majors, men in STEM majors, women in non-STEM majors, and men in non-STEM majors?
**RQ1b:** To what extent does student-reported depression symptom severity differ among women in STEM majors, men in STEM majors, women in non-STEM majors, and men in non-STEM majors?
**RQ2a:** Does student-perceived impact of “anxiety/stress” on academic outcomes differ between men and women and between STEM and non-STEM majors?
**RQ2b:** Does student-perceived impact of “depression/suicidality” on academic outcomes differ between men and women and between STEM and non-STEM majors?

This study specifically focuses on the experiences of cisgender women compared to cisgender men. However, we acknowledge that gender extends beyond these binary categories. While cisgender women [38,39], non-binary individuals [56], and trans individuals [56–58] all demonstrate worse mental health outcomes than cisgender men, qualitative interview studies highlight that non-binary and trans students have distinct experiences during their undergraduate careers that affect their mental health [56,59]. As such, independent analyses on the mental health of LGBTQ+ undergraduates were conducted in a separate investigation.

## Methods

### Ethics statement

The present study is a retrospective study conducted with Healthy Minds Study (HMS) 2022–2023 cohort data. The HMS was initially developed and distributed with an approved Institutional Review Board (IRB) no. HUM00149184. Currently, HMS is an active study overseen by IRB host Advarra for the University of Michigan. Advarra IRB is registered with OHRP and FDA under IRB no. 00000971. All other participating institutions are considered “non-engaged performance sites” which U.S. federal law does not require separate IRB approval unless advised by a participating institution’s own policy or requirement. For all recruited students, written informed consent was acquired. To participate in the survey, students had to be at least 18 years old and seeking a degree from the participating institution. Authors followed HMS protocols for obtaining data authorizations. All data analyzed in this manuscript was anonymized prior to being accessed by authors on July 9, 2024.

### Healthy minds network data

The Healthy Minds Study (HMS) is a national, online survey distributed to U.S. college and university students [60]. Access to the full survey and raw data is available upon request at https://healthymindsnetwork.org/. Access to the processed data and analysis file for R is available in the Supplemental Material, see S1 Data and S1 Analysis. This study analyzes cross-sectional data collected during the Fall 2022 and Spring 2023 semesters. In the Fall of 2022, 46 institutions participated in HMS, and in the Spring of 2023, 89 institutions participated by the end of the academic year. Students were recruited through institutions that opted into the Healthy Minds Network. Institutions included both public and private schools, as well as two-year community colleges and four-year universities. Institutions differed in their approach to surveying students, with some schools offering the survey to the entire student population, while others randomly selected a smaller recruitment pool. Students were invited to participate in the survey via email, and non-responders could receive up to three reminders. All variables analyzed in this dataset were treated as cross-sectional between-subjects data. The average response rate for this cohort is 9%, which is considered typical for online opt-in surveys of college students [61].

### Measures

#### Demographics.

Study participants were asked a suite of demographic questions. Below we outline the demographics used as covariates in our study.

Participants were asked, “What is your gender identity?” and were provided with the following mutually exclusive choices: male, female, trans male/trans man, trans female/trans woman, genderqueer/gender non-conforming, self-identify (other), gender non-binary, or prefer not to respond. For the purposes of this study, participants who identified as “female” were coded as “women” and participants who identified as “male” were coded as “men”. When we refer to “men” and “women” in this paper, we are referring to cisgender men and cisgender women. We chose to exclude participants who identified as “trans male/trans man, trans female/trans woman, genderqueer/gender non-conforming, or self-identify (other), gender non-binary,” because we did not feel it was appropriate to aggregate these identities together into a third category, since their lived experiences are likely very different [56,59]. Instead, independent analyses on the mental health of LGBTQ+ undergraduates were conducted in a separate investigation.

Participants were asked to choose their field of study from a list. Based on the National Science Foundation’s list of research areas [62], the following fields were considered STEM fields: “natural sciences or mathematics,” “social sciences,” “engineering,” “nursing,” “pharmacy,” and “public health.” For the purposes of our research questions, all other fields in the dataset were aggregated into non-STEM: “humanities,” “architecture or urban planning,” “art and design,” “business,” “education, “music, theatre, or dance,” “public policy,” “undecided”, or “other.” Students were asked about their race/ethnicity, with the following options available for single-select: “African American/Black,” “American Indian or Alaskan Native,” “Asian American/Asian,” “Hispanic/Latin(x),” “Native Hawaiian or Pacific Islander,” “Middle Eastern, Arab, or Arab American,” “White,” and “Self-identify (please specify).” For the purposes of this study, Pacific Islanders, Native Americans, and students who selected the self-identify option were excluded due to low sample size. Students also selected to what extent they would describe their current financial status at time of survey, from the following response options: “never stressful,” “rarely,” “sometimes,” “often,” and “always stressful.” This variable was re-coded as an ordinal variable such that “never stressful” equaled 1 and “always stressful” equaled 5. Additionally, participants were asked to report their age and current year in their undergraduate program. Age was treated as a continuous numeric variable, while current year in their undergraduate program was treated as categorical with the following choices: “first-year,” “second-year,” “third-year,” and “fourth-year or greater.”

#### RQ1. To what extent does anxiety and depression symptom severity differ among women in STEM majors, men in STEM majors, women in non-STEM majors, and men in non-STEM majors? *RQ1a. Anxiety symptom severity: Generalized Anxiety Disorder-7 (GAD-7).*

To assess anxiety symptom severity, participants were presented with the GAD-7, a widely used and well-validated self-report survey for assessing anxiety symptom severity in this population [63,64]. The GAD-7 is composed of seven questions regarding anxiety symptom frequency over the last two weeks (e.g., “Over the last 2 weeks, how often have you been bothered by the following problem? Feeling nervous, anxious or on edge.”) Participants responded to each item on a Likert scale ranging from “1=Not at all” to “4=Nearly every day.” Internal consistency calculated for the present study (N = 43,910) was α = .92. GAD-7 mean scores were used to quantify self-reported anxiety.

***RQ1b. Depression symptom severity: Patient Health Questionnaire-9 (PHQ-9)***. To assess depression symptom severity, participants were presented with the PHQ-9, a widely used and well-validated self-report survey for assessing depression symptom severity in this population [65,66]. The PHQ-9 is composed of nine questions probing the frequency of depression symptoms over the last two weeks (e.g., “Over the last 2 weeks, how often have you been bothered by any of the following problems? Little interest or pleasure in doing things”). Participants responded on a Likert scale ranging from “1=Not at all” to “4=Nearly every day.” Internal consistency calculated for the present study (N = 43,910) was α = .90. PHQ-9 mean scores were used to quantify self-reported depression.

#### RQ2. Does student-perceived impact of “anxiety/stress” and “depression/suicidality” on academic outcomes differ between men and women and between STEM and non-STEM majors? *RQ2a. Student-perceived impact of “anxiety/stress” on academic outcomes.*

A subset of universities chose to send an additional auxiliary survey module to their students asking about the effects of mental health on various academic outcomes. This subset of students was given the following matrix question, “In the past year, how has anxiety/stress affected your academic performance?” and invited to select as many options as applied to them including: “I did not experience this [referring to anxiety/stress]” (labeled item 1), “I experienced this but it did not affect my academic performance” (item 2), “I received a lower grade on one or more exams or projects” (item 3), “I received a lower grade in one or more courses” (item 4), “I received an incomplete or dropped one or more courses” (item 5), “I had a significant disruption in research practicum, thesis, or dissertation work” (item 6), and “other” (item 7). Since these items were not considered part of a scale, each response option was treated as a separate single-item measure and coded dichotomously (0 = student did not select, 1 = student selected).

***RQ2b. Student-perceived impact of “depression/suicidality” on academic outcomes***. A subset of universities chose to send an additional auxiliary survey module to their students asking about the effects of mental health on various academic outcomes. This subset of students were also given the following matrix question, “In the past year, how has depression/suicidality affected your academic performance?” and invited to select as many options as applied to them including: “I did not experience this [referring to depression/suicidality]” (labeled item 1), “I experienced this but it did not affect my academic performance” (item 2), “I received a lower grade on one or more exams or projects” (item 3), “I received a lower grade in one or more courses” (item 4), “I received an incomplete or dropped one or more courses” (item 5), “I had a significant disruption in research practicum, thesis, or dissertation work” (item 6), and “other” (item 7). Since these items were not considered part of a scale, each response option was treated as a separate single-item measure and coded dichotomously (0 = student did not select, 1 = student selected).

### Data analysis

The statistical program R version 4.4.1 was used for analyses [67]. The following packages were used: dplyr [68], Hmisc [69], psych [70], forcats [71], ggplot2 [72], jtools [73], and tidyverse [74].

#### RQ1: To what extent does anxiety and depression symptom severity differ among women in STEM majors, men in STEM majors, women in non-STEM majors, and men in non-STEM majors? *RQ1a.*

We used linear regression to assess to what extent the severity of anxiety symptoms differs among students who identified as man or woman in STEM versus non-STEM majors. We regressed students’ GAD-7 scores on their gender, whether they were a STEM or non-STEM major, and the interaction between gender and major. We chose to conduct a regression rather than a t-test to control for race/ethnicity and financial stability, since these demographics have been shown to be predictive of student mental health [75–77]. We also chose to control for age and year in undergraduate career to potentially improve model fit [78,79]. For the categorical variables, the reference groups were non-STEM majors, men, White, and first-year undergraduates. Current financial status was treated as an ordinal variable ranging from 1 to 5, where larger values indicate more financial instability. Age was centered and treated as continuous. [Model: GAD-7 ~ major + gender + STEM major * gender + race/ethnicity + financial stability + age (centered) + year in undergraduate career.]

***RQ1b.*** To assess the extent to which the severity of depression symptoms differs among men and women in STEM versus non-STEM undergraduate majors, we ran a regression with PHQ-9 scores as the outcome variable: [Model: PHD-9 ~ major + gender + STEM major * gender + race/ethnicity + financial stability + age (centered) + year in undergraduate career.]

For both regression analyses (RQ1a on GAD-7; RQ2a on PHQ-9), heteroskedasticity was checked via Q-Q plots and determined to be minimal. Variance inflation factor (VIF) was used to confirm there was minimal multicollinearity (see S1 Tables for exact VIF values.)

Given that we use an interaction term between STEM major and gender as a moderator for both regression models, in a separate set of exploratory analyses, we orthogonalized this term to better control for risk of multicollinearity between this term and the main effects; these separate analyses better accounted for the shared variance between the moderator and the main effects. However, the orthogonalization did not change the effect sizes or the interpretations. Further, the interpretation of these exploratory analyses for both the anxiety and depression models were consistent with the simpler models, which did not use orthogonalization. We report the simpler analyses in the results for ease of interpretation.

#### RQ2: Does student-perceived impact of “anxiety/stress” and “depression/suicidality” on academic outcomes differ between men and women and between STEM and non-STEM majors? *RQ2a.* “Anxiety/stress” chi-square analysis for gender and major.

Using the individual items from *impact of self-reported “anxiety/stress” on academic performance* (see *Measures*), we removed participants who selected only item 1, “I did not experience this,” to create a subset of students who self-reported experiencing anxiety/stress (n _anxiety/stress _= 2,081; 501 were men and 1,580 were women). With this subset, we calculated percentages for items 2–7 of this measure using the number of women who responded “yes” to each item over the total number of women who reported experiencing stress/anxiety. We repeated this process for men. These percentages were generated to create bar graphs. Although all students who responded to items 2, 3, 4, 5, 6 and 7 were included in the subset, item 2 (“I experienced this, but it did not affect my academic performance”) is reported in the text but not in the graphic, and items 3–7 were used in graphic generation.

We conducted one chi-square test to explore the differences between groups of interest for each academic performance item. Pearson’s Chi-squared test with Yates’s continuity correction (to account for the dichotomous nature of the data) was used with the Benjamin-Hochberg procedure (to correct for multiple chi-square tests) to assess whether there was a significant difference in the proportion of women versus men who self-reported that anxiety/stress affected the academic outcomes described in items 2–7.

***RQ2b.* “Depression/suicidality” chi-square analysis for gender and major**. Using the individual items from *impact of self-reported “depression/suicidality” on academic performance* (see *Measures*), the methods for RQ2a for both gender and major were repeated for RQ2b, to investigate the analogous items relatedself-reported depression/suicidality (n _depression/suicidality_ = 1,153 students; of which 226 were men and 927 were women; of which 537 were non-STEM majors and 616 were STEM majors).

## Results

### Participant characteristics

A total of 43,910 students who completed the HMS survey in the 2022–2023 academic year were included in this analysis. Participants were predominantly women (72.1%) and White (65.1%). A summary of participant demographics is available in Table 1.

**Table 1:** Summary of participant demographics.

		*N*	%
Major	STEM	18,957	46.2%
	non-STEM	22,065	53.8%
Gender	Men	12,271	27.9%
	Women	31,639	72.1%
Race	White	25,215	65.1%
	Asian	5,118	13.2%
	Black	4,446	11.5%
	Hispanic	3,332	8.6%
	Middle eastern	647	1.7%
Current Financial Stability	Never stressful	3,570	8.1%
	Rarely stressful	9,274	21.1%
	Sometimes stressful	14,964	34.1%
	Often stressful	9,950	22.7%
	Always stressful	6,108	13.9%
Age	Mean:	20.97	
	Standard deviation:	4.64	
Year in school	First year	11,639	26.8%
	Second year	10,498	24.1%
	Third year	10,597	24.3%
	Fourth year or greater	10,789	24.8%

#### Finding 1a: College women report more severe symptoms of anxiety compared to college men.

**Fig 1:**
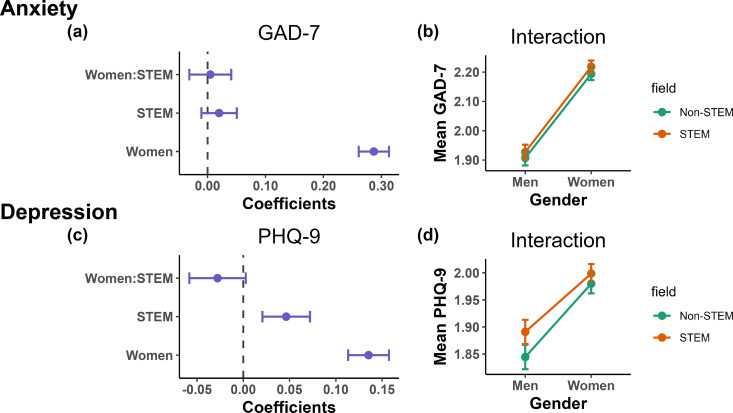
Forest and interaction plots of gender and major for anxiety and depression symptom severity. Forest plot for severity of (A) symptoms of anxiety and (C) symptoms of depression regressed on gender and major. Plots of interaction between gender and major with regard to (B) anxiety symptom severity and (D) depression symptom severity. (A,C) Regression coefficients representing whether students’ (A) anxiety symptom severity and (C) depression symptom severity is predicted by gender, major, and the interaction between the two variables. Estimated confidence intervals in purple that do not cross the dashed line at x = 0 indicate a significant difference. Points to the right of the line indicate that the demographic group reports greater symptom severity compared with the reference group; those to the left indicate that the group reports lesser symptom severity. Reference groups are non-STEM majors and men. Racial identity, current financial stress, age, and year of undergrad are controlled in the models but not shown here. (B,D) This is the interaction term between gender and STEM major plotted for (B) anxiety symptom severity and (D) depression symptom severity. For the interaction plots, the lack of any crossover between the green and orange lines indicates no significant interaction.

When controlling for race/ethnicity, current financial stress, age, and year in undergraduate career, women reported more severe symptoms of anxiety compared to men (*p* < 0.001; GAD-7 average range 1–4; *M*_Women_* *= 2.25, *SD*_Women_ = 0.831; *M*_Men_* *= 1.91, *SD*_Men_ = 0.779; Fig 1A). However, anxiety symptom severity was not significantly different between STEM and non-STEM majors (*p* = 0.18; *M*_STEM_* *= 2.16, *SD*_STEM_ = 0.827; *M*_non-STEM_* *= 2.14, *SD*_non-STEM_ = 0.835; Fig 1B). The interaction between gender and major was also not significant (*p* = 0.79; Fig 1A,B), suggesting that the gender difference in anxiety symptom severity was neither exacerbated nor alleviated among STEM majors compared to non-STEM majors. See S1 Tables for the full regression output, relevant descriptive statistics, and correlation matrices.

#### Finding 1b: College STEM students and women report more severe symptoms of depression compared to their respective counterparts.

When controlling for race/ethnicity, current financial stress, age, and year in undergraduate career, women reported more severe symptoms of depression compared to men (*p* < 0.001; PHQ-9 average range 1–4; *M*_Women_* *= 2.04, *SD*_Women_ = 0.703; *M*_Men_* *= 1.86, *SD*_Men_ = 0.688; Fig 1C). Further, STEM majors reported more severe symptoms of depression compared to non-STEM majors (*p* < 0.001; *M*_STEM_* *= 2.00, *SD*_STEM_ = 0.701; *M*_non-STEM_* *= 1.98, *SD*_non-STEM_ = 0.707; Fig 1D). The interaction between gender and major was not significant (*p* = 0.07; Fig 1C,D), suggesting that the gender difference in depression symptom severity was neither exacerbated nor alleviated among STEM majors compared to non-STEM majors. See S1 Tables for the full regression output, relevant descriptive statistics, and correlation matrices.

#### Finding 2a: A greater proportion of women and STEM students perceived that “anxiety/stress” affected their academic performance on exams and projects compared to their respective counterparts.

**Fig 2:**
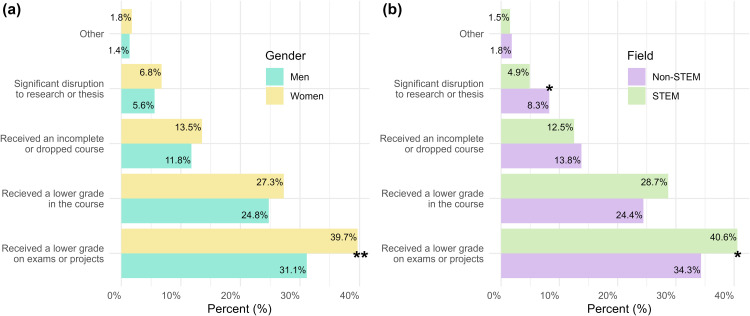
The ways in which anxiety/stress affects student academic performance by gender and major. Double bar graph depicting the proportion of (A) women and men and (B) STEM and non-STEM majors who selected specific ways in which anxiety/stress affects them their academic performance. Percentages were calculated for each item (e.g., received a lower grade in the course) by dividing the number of students in a specific group (e.g., women; STEM) who selected that item over the total subsample of those students (e.g., n_women_ = 1,580; n_STEM major_ = 1,102) that reported anxiety/stress. Significance of the corresponding chi-square is indicated with asterisks such that, **p* *> 0.05, *p < 0.05, **p < 0.01, ***p < 0.001. For (A), more women reported receiving a lower grade on an exam or project (*p-adjusted (Benjamini-Hochberg procedure) *= 0.004). For (B), more non-STEM majors reported disruptions to research or theses (*p-adjusted* = 0.012); more STEM majors reported receiving a lower grade on exams or projects (*p-adjusted* = 0.012).

Undergraduates most commonly selected that they perceive their anxiety/stress contributed to them receiving a lower grade on one or more exams or projects; this consequence was endorsed by a greater proportion of women compared to men (*X*^*2*^ (1, *N* = 2,081) = 11.48, *p* = 0.001,* p-adjusted (Benjamini-Hochberg procedure) *= 0.004, φ = 0.1), as well as STEM students compared to non-STEM students (*X*^*2*^ (1, *N* = 2,081) = 8.34, *p* = 0.004, *p-adjusted* = 0.012, φ = 0.06, **Fig 2**). In contrast, a greater proportion of non-STEM students reported perceiving a significant disruption in research, practicum, thesis, or dissertation work as a result of their anxiety/stress compared to STEM students (*X*^*2*^ (1, *N* = 2,081) = 9.18, *p* = 0.002, *p-adjusted* = 0.012, φ = 0.07). Of students who self-reported experiencing anxiety/stress, 45.5% reported that their anxiety did not affect their academic performance. The percentage of men who did not report any academic consequences from anxiety/stress (51.3%) was significantly higher than the percentage of women who did not report any academic consequences from anxiety/stress (43.5%) (*X*^*2*^ (1, *N* = 2,081) = 8.91, *p* = 0.003, *p-adjusted* = 0.008, φ = 0.09). See S1 Tables for all chi-square analyses and additional descriptive statistics.

#### Finding 2b: There were no significant differences between the proportion of women and men or STEM and non-STEM majors who perceived that “depression/suicidality” affected specific academic outcomes.

**Fig 3:**
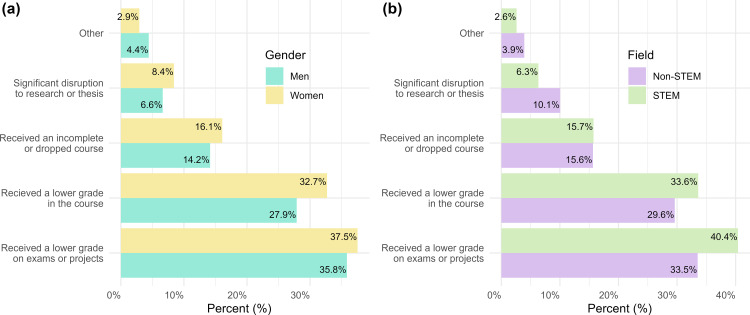
The ways in which depression/suicidality affects student academic performance by gender and major. Double bar graph depicting the proportion of (A) women and men and (B) STEM and non-STEM majors who selected specific ways in which depression/suicidality affects them in the context of academia. Percentages were calculated for each item (e.g., received a lower grade in the course) by dividing the number of students in a specific group (e.g., women; STEM major) who selected that item over the total subsample of those students (e.g., n_women_ = 927; n_STEM major_ = 616) that reported depression/suicidality (N = 1,153).

We found no differences in the proportion of men and women (Fig 3A) or STEM and non-STEM majors (Fig 3B) who reported each of the specific ways that they perceive depression/suicidality affects them academically. Further, there were neither gender nor major differences in the proportion of students who experienced depression/suicidality who reported that they do not perceive it to affect their academic performance. See S1 Tables for all chi-square analyses and additional descriptive statistics.

## Discussion

This study provides the first national investigation of collegiate gender mental health disparities in STEM fields compared to non-STEM fields. In alignment with prior literature demonstrating that women tend to report greater mental health struggles than men [38,39], our study concluded that women report significantly more severe symptoms of anxiety and depression compared to men, both within and outside of STEM majors. However, a student’s major did not moderate the relationship between anxiety or depression symptom severity and gender, meaning that the gender differences in anxiety and depression symptom severity were neither magnified nor minimized in STEM majors compared to non-STEM majors. This finding is surprising considering the unique challenges that women in STEM face, including lower feelings of belonging, lower self-efficacy, and lower science identity compared to men [40].

We found that STEM students reported more severe depression symptoms compared to their non-STEM counterparts. However, the difference between STEM and non-STEM anxiety symptom severity was not significant. It is possible that undergraduates who already struggle with depression are more likely pursue STEM majors. Some evidence suggests that youth intelligence may be associated with later-in-life struggles with mental health [80]. Yet, it is unknown whether students with higher aptitudes are more inclined to choose a STEM major over a non-STEM major; as such, this hypothesis warrants further testing. There is also evidence that individuals with tendencies to be highly fastidious and self-controlled are more likely to be scientists than non-scientists [81]; extreme levels of these traits may result in maladaptive perfectionism, which can contribute to poor mental health [82,83]. Additionally, the notoriously chilly [23,24], competitive [3,4], and unwelcoming [7,25] environments of STEM spaces may contribute to depression symptoms [22,27,84]. Notably, there have been documented efforts to identify and alleviate sources of anxiety specifically in the context of STEM courses [85–87], but fewer efforts focused on depression. As such, these results suggest STEM fields may benefit from studies identifying and addressing aspects of STEM fields that worsen symptoms of depression among undergraduates.

In this study, we also examined how students perceive various academic outcomes are impacted by their “anxiety/stress” and “depression/suicidality,” respectively. Encouragingly, of the students who experienced anxiety/stress and depression/suicidality in our sample, there were few differences in the proportion of women compared to men and between STEM majors compared to non-STEM majors who indicated academic consequences associated with their anxiety/stress or depression/suicidality. However, women were significantly more likely than men to perceive that their self-reported anxiety/stress had a negative effect on their performance on exams or projects. This aligns with one study of undergraduates taking biology courses, which found that for women, but not men, test anxiety negatively impacted exam performance [88]. In the current study, STEM majors were also more likely than non-STEM majors to perceive a negative impact of anxiety/stress on their performance on exams or projects. While it is possible that STEM exams or projects are more likely to evoke feelings of anxiety/stress and the associated consequences [88,89] than non-STEM exams or projects, further investigation is needed to understand the reasoning for this finding. Interestingly, non-STEM majors were more likely to perceive that anxiety/stress caused a significant disruption in their research or thesis. This is counterintuitive given that conducting undergraduate research is more common in STEM fields than in non-STEM fields. We posit that there may be less formal mentoring and psychosocial support for non-STEM majors pursuing research [90,91], and thus, non-STEM students are more likely to perceive that experiencing anxiety/stress has greater consequences on their research.

Our results highlight that simply looking at disparities in rates of psychopathology is not enough. Future research should examine disparities in the effects of psychopathology on academic success, regardless of whether differences in rates of anxiety and depression exist. Universities can consider this as they develop, advertise, and implement mental health resources for undergraduates.

### Limitations and future directions

This study was constrained by how the data were collected in the Healthy Minds Study. For example, institutions differed in their approach to surveying students, with some schools offering the survey to the entire student population, while other institutions randomly selected a smaller sample of students to survey. Further, questions about the impact of mental health on academic performance were double-barreled, in which participants were asked to self-report about their (verbatim, as seen by participant) “depression/suicidality” and “stress/anxiety.” While suicidality is a symptom of depression, not all students who experience depression experience suicidality [14]. Similarly, not all students who experience stress experience anxiety [13]. As such, future research should aim to disentangle the impact of depression and suicidality, as well as stress and anxiety, on student academic outcomes. The survey also relied on students’ self-reported perceptions of how their mental health impacted their academic experiences. However, students’ perceptions may not accurately reflect the effect of their mental health on their academic experiences and recall bias can result in inaccurate memories [92]. To address this limitation, future studies should examine whether academic outcomes, such as exam scores, differ between women and men with similar symptoms of anxiety and/or depression.

## Conclusion

In a national sample of 43,910 undergraduates, women reported more severe symptoms of anxiety and depression compared to men. STEM students reported more severe depression symptoms compared to non-STEM students. However, being a STEM major did not exert an exacerbating or buffering effect on the symptom severity of anxiety or depression in women compared to men. Both women and STEM majors were more likely than their respective counterparts to report perceiving a negative impact of anxiety/stress on exam performance. These findings suggests that as universities strive to improve mental health among their undergraduates, they should acknowledge that college women and STEM students exhibit more severe depression symptoms than their respective counterparts, and the impact of “anxiety/stress” on exam performance may disproportionately disadvantage women and STEM students.

## Supporting information

S1 Table Supporting information tables. (PDF)

S1 Data De-identified processed data used in the analysis file. (CSV)

S1 Analysis Analysis coding script for the statistical software R. (R)
