# Supplementary material for: STEM, gender, and mental health: Understanding depression and anxiety in a national undergraduate sample
Source: PLOS Ment Health. 2025 Aug 26;2(8):e0000364. doi: 10.1371/journal.pmen.0000364 (PMC12798185; doi:10.1371/journal.pmen.0000364)
Supplement: S1 Table — (PDF) [file pmen.0000364.s001.pdf]

Supplemental Material

Plos Mental Health
Pigart et al.

Supplemental Material for
STEM, gender, and mental health: understanding depression and anxiety in a national undergraduate sample.

Authors: C Jynx Pigart,[a] Katherine A. Cohen, MA,[b] Riley McDanal, MA,[c] Nicholas Eaton, PhD,[c] Jessica L. Schleider, PhD,[b] & Katelyn Cooper, PhD.[a]

[a]*School of Life Sciences, Arizona State University*
[b]*Department of Medical Social Sciences, Northwestern University*
[c]*Department of Psychology, Stony Brook University*

This supplement document contains the following:

Item	Page number
S1A Table. Descriptive statistics and correlation matrices relevant to regressions.	2
S1B Table. Summary of regression analyses for anxiety and depression regression models.	3
S1C Table. Results of chi-square tests: anxiety affecting academic performance by gender & field.	4
S1D Table. Results of chi-square tests: depression affecting academic performance by gender & field.	5
S1E Table. Descriptive information of anxiety/stress items affecting academic performance.	6
S1F Table. Descriptive information of depression/suicidality items affecting academic performance.	7

S1A Table. Descriptive statistics and correlation matrices relevant to regressions.

Descriptive statistics.

			Depression (PHQ-9)		Anxiety (GAD-7)	
**Gender**	**Field**	**N***	**Mean**	**Sd**	**Mean**	**Sd**
Men	Non-STEM	5770	1.854	0.692	1.913	0.791
Men	STEM	5928	1.876	0.686	1.904	0.767
Women	Non-STEM	13187	2.035	0.706	2.24	0.835
Women	STEM	16137	2.047	0.7	2.258	0.828

**Combined (all)**	**N***	**Mean**	**Sd**	**Mean**	**Sd**
Non-STEM	18957	1.98	0.707	2.14	0.835
STEM	22065	2.00	0.701	2.16	0.827

**Combined (all)**	**N***	**Mean**	**Sd**	**Mean**	**Sd**
Men	12271	1.86	0.688	1.91	0.779
Women	31639	2.04	0.703	2.25	0.831

** Participants who declined to state their major (N = 2,888) were excluded from regression analyses.*

Correlation matrices.

	**PHQ**	**GAD**	**Age (centered)**	**Financial stability**
**PHQ**				
**GAD**	0.742			
**Age (centered)**	-0.026	-0.032		
**Financial stability**	0.342	0.309	0.136	
**Year in school***	0.014	0.033	0.302	0.097

Note. All correlations presented *significant to p < .01.*

** Year in school was treated as a categorical variable in regression analyses, and converted to numeric for this correlation matrix only to convey the relationships with other variables.*

S1B Table. Summary of regression analyses for anxiety and depression regression models.

	Regression model outcome		
	Anxiety (GAD-7)	Depression (PHQ-9)	Variance inflation factor (VIF)
**(Intercept)**	1.20 ***<br>(0.02)	1.18 ***<br>(0.01)	
**Women**	0.29 ***<br>(0.01)	0.14 ***<br>(0.01)	2.10
**STEM**	0.02<br>(0.02)	0.05 ***<br>(0.01)	3.49
**Current financial stress**	0.23 ***<br>(0.00)	0.21 ***<br>(0.00)	1.07
**Asian**	-0.10 ***<br>(0.01)	0.03 **<br>(0.01)	1.06
**Black**	-0.17 ***<br>(0.01)	-0.04 ***<br>(0.01)	1.06
**Hispanic**	-0.09 ***<br>(0.02)	0.00<br>(0.01)	1.06
**Middle Eastern**	0.04<br>(0.03)	0.09 **<br>(0.03)	1.06
**Fourth-year+ student**	0.07 ***<br>(0.01)	0.02<br>(0.01)	1.11
**Second-year+ student**	0.09 ***<br>(0.01)	0.05 ***<br>(0.01)	1.11
**Third-year+ student**	0.07 ***<br>(0.01)	0.03 ***<br>(0.01)	1.11
**Age (centered)**	-0.01 ***<br>(0.00)	-0.01 ***<br>(0.00)	1.12
**Women:STEM (interaction)**	0.00<br>(0.02)	-0.03<br>(0.02)	4.68
**N**	34295	34642	
**R^2**	0.13	0.13	

Table is presented with each predictor as follows: Coefficient estimate, significance, then standard errors, which are reported in parenthesis underneath the corresponding coefficient. Standard errors are heteroskedasticity robust. VIF values were identical for both models.

*** $p < 0.001$; ** $p < 0.01$; * $p < 0.05$.

S1C Table. Results of chi-square tests: anxiety affecting academic performance by gender & field.

Binary demographic	Multi-select option^	X^2	φ Effect Size	p-value^^	BH p-value
**Gender**	2	8.91	0.09	0.003	0.008**
	3	11.48	0.10	0.001	0.004**
	4	1.12	0.03	0.291	0.486
	5	0.89	0.03	0.344	0.486
	6	0.69	0.02	0.405	0.486
	7	0.14	0.01	0.712	0.712
**STEM status**	2	1.73	0.03	0.188	0.282
	3	8.34	0.06	0.004	0.012*
	4	4.6	0.05	0.032	0.064
	5	0.62	0.02	0.43	0.516
	6	9.18	0.07	0.002	0.012*
	7	0.12	0.01	0.724	0.712

Degrees of freedom for all chi squares are equal to 1. Pearson's Chi-squared test with Yates' continuity correction was used for all analyses. P-values are rounded to three decimal places. The sample size was filtered such that any participant who selected at least one Option 2-7* was counted a single time. The sample size for Gender chi-squares is N = 2,081 students (501 men, 1,580 women). The sample size for Field chi-squares is N = 2,081 students (979 Non-STEM, 1102 STEM). Significance: $p > 0.05$, $*p < 0.05$, $**p < 0.01$, $***p < 0.001$. For effect size, a value of $\varphi = 0.1$ is considered to be a small effect, 0.3 a medium effect, and 0.5 a large effect.

^Option 1 was selected if a student did not experience anxiety, which was filtered from these analyses for the purpose of our research questions. Option 2 was selected if a student experienced anxiety, but it did not affect their academic performance. Option 3 was selected if they experienced that their anxiety resulted in a lower grade on one or more exams or projects. Option 4 was selected if a student received an overall lower grade in one or more courses as a result of their anxiety. Option 5 was selected if a student received an incomplete or dropped one or more courses as a result of their anxiety. Option 6 was selected if a student experienced significant disruption in research, practicum, thesis, or dissertation work. Option 7 was selected if a student had other experiences that affected their academic performance as a result of their anxiety. Students were invited to select all that applied to them.

^^ These are the initial p-values that are not corrected for multiple tests. The Benjamini-Hochberg (BH) procedure was then used, which is a statistical method that controls the rate of false positives in multiple hypothesis tests.

S1D Table. Results of chi-square tests: depression affecting academic performance by gender & field.

Binary demographic	Multi-select option^	X^2	φ Effect Size	p-value^^	BH p-value
**Gender**	2	0.09	0.01	0.767	0.767
	3	0.16	0.01	0.691	0.767
	4	1.72	0.04	0.189	0.767
	5	0.37	0.02	0.544	0.767
	6	0.55	0.02	0.457	0.767
	7	0.9	0.03	0.344	0.767
**STEM Status**	2	0.52	0.02	0.471	0.565
	3	5.56	0.05	0.018	0.082
	4	1.93	0.03	0.164	0.329
	5	0	0.00	1	1
	6	4.88	0.05	0.027	0.082
	7	1.2	0.02	0.274	0.411

Degrees of freedom for all chi squares are equal to 1. Pearson's Chi-squared test with Yates' continuity correction was used for all analyses. P-values are rounded to three decimal places. The sample size was filtered such that any participant who selected at least one Option 2-7* was counted a single time. The sample size for Gender chi-squares is N = 1,153 students (226 men, 927 women). The sample size for Field chi-squares is N = 1,153 students (537 Non-STEM, 616 STEM). Significance: $p > 0.05$, *$p < 0.05$, **$p < 0.01$, ***$p < 0.001$. For effect size, a value of φ = 0.1 is considered to be a small effect, 0.3 a medium effect, and 0.5 a large effect.

^Option 1 was selected if a student did not experience depression, which was filtered from these analyses for the purpose of our research questions. Option 2 was selected if a student experienced depression, but it did not affect their academic performance. Option 3 was selected if they experienced that their depression resulted in a lower grade on one or more exams or projects. Option 4 was selected if a student received an overall lower grade in one or more courses as a result of their depression. Option 5 was selected if a student received an incomplete or dropped one or more courses as a result of their depression. Option 6 was selected if a student experienced significant disruption in research, practicum, thesis, or dissertation work. Option 7 was selected if a student had other experiences that affected their academic performance as a result of their depression. Students were invited to select all that applied to them.

^^ These are the initial p-values that are not corrected for multiple tests. No significance was found after using the Benjamini-Hochberg (BH) procedure, a statistical method that controls the rate of false positives in multiple hypothesis tests.

S1E Table. Descriptive information of anxiety/stress items affecting academic performance.

**Mental health aspect**	**Type of academic performance affected**	**Gender**	**Field**	**N**	**N affected (responded yes)**	**% affected**
Anxiety /stress	Disrupted research or thesis	Men	Non-STEM	266	18	6.8
		Men	STEM	235	10	4.3
		Women	Non-STEM	713	63	8.8
		Women	STEM	867	44	5.1
	Experienced this; did not impact academic performance	Men	Non-STEM	266	140	52.6
		Men	STEM	235	117	49.8
		Women	Non-STEM	713	320	44.9
		Women	STEM	867	368	42.4
	Lower grade (final grade)	Men	Non-STEM	266	62	23.3
		Men	STEM	235	62	26.4
		Women	Non-STEM	713	177	24.8
		Women	STEM	867	254	29.3
	Lower grade (on an exam or project)	Men	Non-STEM	266	77	28.9
		Men	STEM	235	79	33.6
		Women	Non-STEM	713	259	36.3
		Women	STEM	867	368	42.4
	Other	Men	Non-STEM	266	2	0.8
		Men	STEM	235	5	2.1
		Women	Non-STEM	713	16	2.2
		Women	STEM	867	12	1.4
	Received incomplete or dropped course	Men	Non-STEM	266	36	13.5
		Men	STEM	235	23	9.8
		Women	Non-STEM	713	99	13.9
		Women	STEM	867	115	13.3

S1F Table. Descriptive information of depression/suicidality items affecting academic performance.

Mental health aspect	Type of academic performance affected	Gender	Field	N	N affected (responded yes)	% affected
Depression /suicidality	Disrupted research or thesis	Men	Non-STEM	113	10	8.8
		Men	STEM	113	5	4.4
		Women	Non-STEM	424	44	10.4
		Women	STEM	503	34	6.8
	Experienced this; did not impact academic performance	Men	Non-STEM	113	46	40.7
		Men	STEM	113	48	42.5
		Women	Non-STEM	424	178	42
		Women	STEM	503	195	38.8
	Lower grade (final grade)	Men	Non-STEM	113	32	28.3
		Men	STEM	113	31	27.4
		Women	Non-STEM	424	127	30
		Women	STEM	503	176	35
	Lower grade (on an exam or project)	Men	Non-STEM	113	38	33.6
		Men	STEM	113	43	38.1
		Women	Non-STEM	424	142	33.5
		Women	STEM	503	206	41
	Other	Men	Non-STEM	113	6	5.3
		Men	STEM	113	4	3.5
		Women	Non-STEM	424	15	3.5
		Women	STEM	503	12	2.4
	Received incomplete or dropped course	Men	Non-STEM	113	16	14.2
		Men	STEM	113	16	14.2
		Women	Non-STEM	424	68	16
		Women	STEM	503	81	16.1
